# Integrative Proteome Analysis Revels 3-Hydroxybutyrate Exerts Neuroprotective Effect by Influencing Chromatin Bivalency

**DOI:** 10.3390/ijms24010868

**Published:** 2023-01-03

**Authors:** Xin-Liang Zhu, Huan Du, Lei-Lei Wang, Er-Ling Hu, Ning Li, Hai-Xia Lu, Guo-Qiang Chen, Xiao-Yun Lu

**Affiliations:** 1 Key Laboratory of Biomedical Information Engineering of the Ministry of Education, School of Life Science and Technology, Xi’an Jiaotong University, Xi’an 710049, China; 2 Division of Life Science, Hong Kong University of Science and Technology, Hong Kong SAR 999077, China; 3 Institute of Neurobiology, School of Basic Medical Sciences, Xi’an Jiaotong University Health Science Center, Xi’an 710049, China; 4 MOE Key Laboratory of Bioinformatics, Department of Biological Science and Biotechnology, School of Life Sciences, Tsinghua-Peking Center for Life Sciences, Tsinghua University, Beijing 100084, China

**Keywords:** 3-hydroxybutyrate, proteomics, transcriptomics, chromatin bivalency, neuroprotection

## Abstract

3-hydroxybutyrate (3OHB) has been proved to act as a neuroprotective molecule in multiple neurodegenerative diseases. Here, we employed a quantitative proteomics approach to assess the changes of the global protein expression pattern of neural cells upon 3OHB administration. In combination with a disease-related, protein-protein interaction network we pinpointed a hub marker, histone lysine 27 trimethylation, which is one of the key epigenetic markers in multiple neurodegenerative diseases. Integrative analysis of transcriptomic and epigenomic datasets highlighted the involvement of bivalent transcription factors in 3OHB-mediated disease protection and its alteration of neuronal development processes. Transcriptomic profiling revealed that 3OHB impaired the fate decision process of neural precursor cells by repressing differentiation and promoting proliferation. Our study provides a new mechanism of 3OHB’s neuroprotective effect, in which chromatin bivalency is sensitive to 3OHB alteration and drives its neuroprotective function both in neurodegenerative diseases and in neural development processes.

## 1. Introduction

The ketone body 3-hydroxybutyrate (3OHB) can be metabolized to fulfill the major portion of the brain’s alternative energy source under certain conditions [1]. It has been demonstrated that 3OHB can exert significant beneficial effects against extracellular stress and enhance the synaptic plasticity of neurons [2]. The normal adult brain depends on the continuous supply of glucose from blood flow rather than exogenous fatty or amino acids to carry out appropriate functions, especially for neurons [3]. The largest part of the 3OHB utilized by the brain is supplied by the liver via the blood stream, mostly during physiological fasting and vigorous exercise [4]. It has also been observed that the external administration of 3OHB or a ketogenic diet can be beneficial for the treatment of certain neurodegenerative diseases, suggesting that ketone bodies might have clinical therapeutic potential for control the progression of Alzheimer’s disease (AD), Parkinson’s disease (PD), Huntington’s disease (HD), and amyotrophic lateral sclerosis (ALS) [5,6,7]. In particular, ketone bodies have been indicated to reduce amyloid neurotoxicity and related pathologies, improving synaptic plasticity and cognitive performance in an AD model [8]. Preclinical research has also discovered that the incorporation of 3OHB was able to increase longevity, reduce motor debility, and inhibit striatal histone deacetylation in transgenic HD model R6/2 mice [9]. Additionally, ketogenic diets were able to alter the development of the clinical and biological character of the G93A SOD1 transgenic ALS mouse model [10].

**Figure 1:**
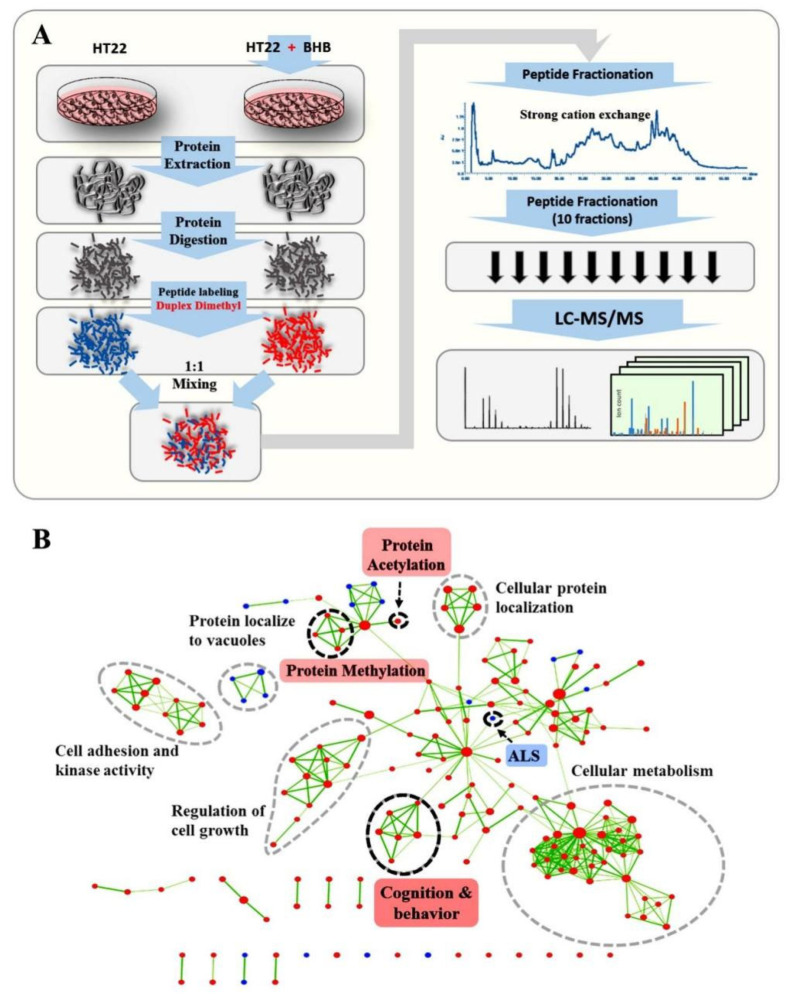
**Proteomics Workflow and GSEA Analysis**. (**A**) HT22 cells were treated with 0.2 mM of β-hydroxybutyrate (3OHB) and a mock solution, respectively. Cells were then processed for MS-based dimethyl-labeling quantitative proteomics analysis. (**B**) GSEA was applied to extract an enriched gene ontology map from the proteomics data. As indicated by the pink tag, pathways related to protein acetylation, neurodegenerative disease, ALS and cognition were especially highlighted, which was in good agreement with previous studies. At the same time, biological processes related to protein methylation also showed sensitive responses to 3OHB administration in normal neurons. Red dot represents up-regulated processes and pathways, blue dot represents down-regulated processes and pathways, the green line indicates shared proteins between connected processes and pathways, where thickness is proportional to the number of shared proteins.

We first applied a single-variant proteomics study on normal mouse neurons. We then conducted a study of widespread changes in several molecular layers in a progressive manner and revealed the dynamic regulation processes of information flow by assessing proteomic changes integrated with changes of the epigenome and transcriptome. Common dysregulation of neurological disorders with a connection to 3OHB was elucidated by leveraging networks of differentially co-expressed gene pairs and physical protein interactions, which revealed several interesting hub markers: H3K27me3, DNMT1, DNMT3A, H2AK118bhb1, and H2AK119ub1. Lastly, we analyzed the truncated sub-networks of epigenetic regulators and attempted to pinpoint and validate the correlations of this sub-network with recent evidence on changes of gene expression patterns upon 3OHB administration (Figure 1A).

Our findings provide compelling evidence that the histone-methylation epigenomic network may indeed play an important role in 3OHB administration and further mediate its neuroprotective effects.

## 2. Results

### 2.1. Proteomics Assessment of the Effects of 3OHB Treatment in Neuronal Cells

Considering the fact that there are varying amounts of endogenous 3OHB and other fatty acids in different animal tissues, or even different regions of the tissues, it is warranted to study normal neurons rather than the entire brain or separate tissues in order to evaluate the proteomic response to 3OHB. The 3OHB source was limited at a basal level from serum in the culture medium during HT22 cell culturing for proteomics sample preparation. Considering that the peak concentration after prolonged fasting or during a ketogenic diet may only reach 3 to 4 mM, and the fact that 3OHB barely directly penetrates the BBB (brain-blood-barrier), we assumed a 3OHB concentration in the hippocampal extracellular fluid of 0.2 mM for the proteomic study [11,12]. This way, we were able to assess the physiologically relevant response of neurons to 3OHB treatment rather than responses that only occur at high concentrations that are unrealistic in vivo.

We then used tandem mass spectrometry LC-MS/MS to systematically quantitate the protein expression profiles which ultimately determine the cellular phenotypes [13]. To facilitate the comparison of proteome-wide 3OHB effects without interference from other metabolites, all experiments were conducted using the same HT22 cell line, grown under the same culture conditions, with each set of MS sample preparation being performed in biological quadruplicate. In total, 40,358 peptides were identified, corresponding to 5454 proteins identified with two or more peptides per protein (submitted to ProteomeXchange via PRIDE; accession code: PXD008053). The data were further filtered to include only those proteins detected and quantified in at least three out of four replicates. This produced a high-quality dataset comprising 3748 proteins, which were further used to evaluate changes of their abundance for downstream analysis (Supplementary Table S1).

### 2.2. Unbiased Gene Set Enrichment Analysis Identified Pathways That Were Highly Enriched amongst Proteins Altered in Abundance after 3OHB Treatment

To elucidate the entire pattern of proteome alteration in HT22 cells upon 3OHB treatment, we used an alternative approach, named unbiased gene set enrichment analysis [14]. All of the identified and quantified proteins were considered as contributors to specific biological processes and pathways without setting a specific cutoff for significant changes. As shown in Figure 1B, several key biological processes and pathways were relatively enriched among all proteome perturbation patterns. The upregulation of protein acetylation was in agreement with previous research on the HDAC inhibitory function of 3OHB [15,16]. The proteins in this pathway, which includes mostly histone acetylation enzymes and those connected to cellular metabolism and cell growth, were also upregulated upon 3OHB treatment, which was expected and has been reported before [2,17]. Interestingly, pathways related to ALS were also highlighted in this analysis, which directly correlated with previous research [10]. Particularly, processes related to protein methylation formed a special cluster in this analysis. Interestingly, it has recently been reported that the lysine 4 trimethylation level of histone 3 was altered by a ketogenic diet in a transgenic model of Kabuki syndrome [18]. However, there are few other studies on the direct correlation of 3OHB and histone methylation.

### 2.3. An Analysis of the Disease-Related Protein-Protein Interaction Network Indicated That H3K27me3 Is a Hub Marker of the Neuronal Response to 3OHB

**Figure 2:**
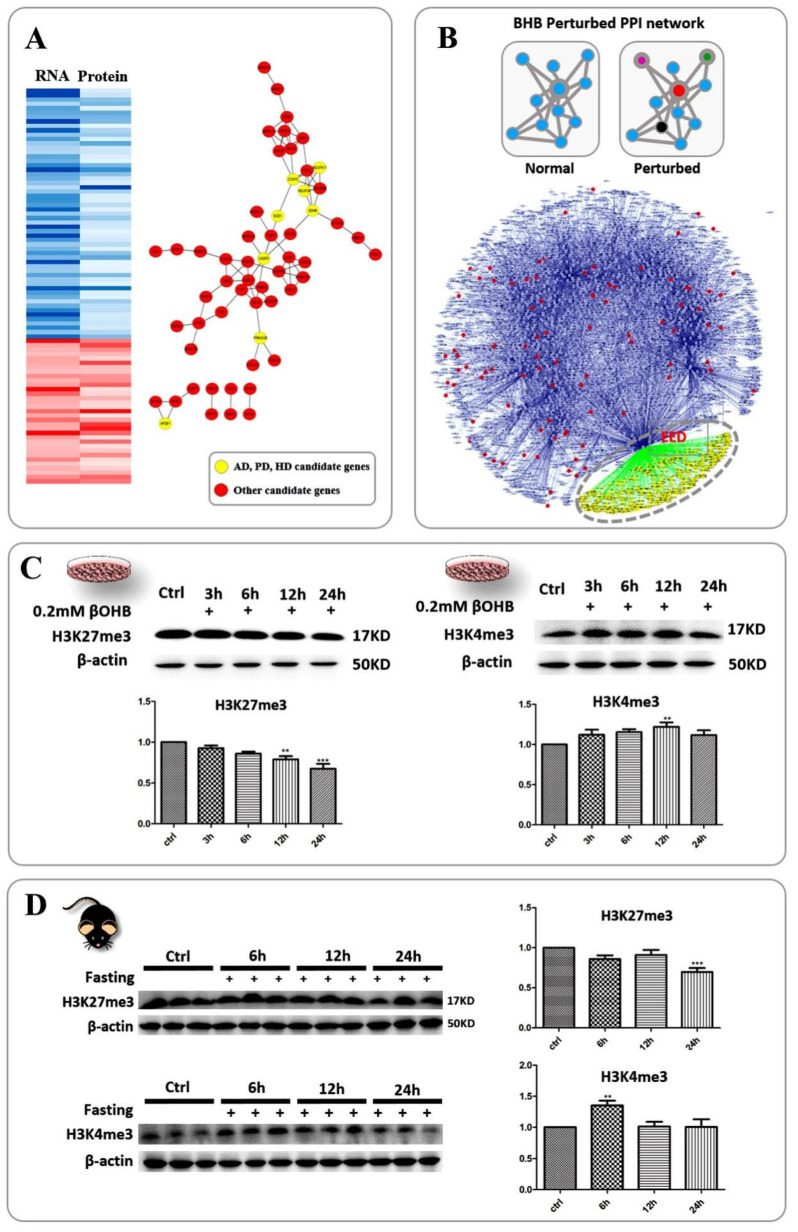
**Integrative analysis of the proteomics dataset pinpointed H3K27me3 and H3K4me3 as hubs.** (**A**) Proteomics data from our study and transcriptomic data from references overlapped. Left, genes that showed a consistent increase or decrease in both RNA and protein levels are highlighted by red and blue columns, respectively; right, the network that was generated using the STRING database by loading consistently changed genes as seeds which constitute nodes of a protein interaction network. Among them, yellow nodes represent genes related to neurodegenerative disease like AD, PD and HD. (**B**) Up, the protein-protein interaction model used to identify hub proteins. Down, the protein-protein interaction network, was seeded with AD, PD, HD, Epilepsy, ASD, and ALS candidate proteins identified in our dataset (Supplementary Table S2); highlighted by red nodes. Blue nodes indicate intermediate protein-protein interactions between seed genes. Green nodes represent proteins connected to the top hub protein of high degree and/or betweenness centrality. (**C**) Western blot analysis (*n* = 3), left, revealed that 3OHB induced a significant reduction of H3K27me3 levels in the HT22 cells upon long-term treatment (at 12 h (*p* < 0.01) and 24 h (*p* < 0.001)); right, the levels of H3K4me3 in the HT22 cells were increased after 12 h of treatment (*p* < 0.01). (**D**) Western blot analysis (*n* = 6), left, revealed that fasting induced a similar pattern of H3K27me3 level reduction in the brains of C67 mice at 24 h (*p* < 0.001) comparable to that of HT22 cells; right, H3K4me3 levels showed an earlier response at 6 h of fasting (*p* < 0.01) than what was observed upon 3OHB treatment in HT22 cells. Data are mean ± SEM *n* = 3 (**C**); Data are mean ± SEM *n* = 6 mice per group (**D**), one-way ANOVA, Tukey’s test, ** means *p* < 0.01 while *** means *p* < 0.001 (**C**,**D**).

Considering the fact that 3OHB has the potential to protect neurons from neurodegenerative diseases, we included data from reference transcriptomes to identify genes that respond to 3OHB at both the RNA and protein levels (Figure 2A, left). The protein interaction and gene annotation information was obtained by loading these seed genes into the STRING database. As shown in Figure 2A, right, 3 typical pre-disease processes were also indicated in the network by yellow nodes. Using the data combination as described above, we examined our data for disease-related proteins whose expression was altered during 3OHB administration. After searching the available disease databases, we compiled a list of overlapping targets between our proteomics dataset and the epilepsy, AD, PD, HD, ASD and ALS databases (see Methods, Supplementary Table S2).

To explore this further, we examined the protein interaction networks and signaling pathways. We hypothesized that if disease-related alterations of protein abundance were able to cause vulnerability of neurons, 3OHB administration might be able to induce alterations of several key hubs which are correlated with disease-associated proteins. It was also expected that these hub proteins play critical roles in the pathogenesis of neurodegenerative diseases, and, thus, might mediate the multiple protective functions of 3OHB.

Based on these hypotheses and previous knowledge, we generated a custom database encompassing all six neuronal disorders as the background dataset, and the network of potential protein-protein interactions (PPIs) among proteins related to the six disorders, as quantified based on our MS dataset. To do this, we first generated a PPI subnetwork for each disorder by mapping its related proteins (78 in AD, 118 in PD, 46 in HD, 39 in ALS, 124 in ASD and 60 in Epilepsy) with reference PPI networks as described in the methods part. Sub-networks were then built by increasing the number of neighbors to the seed proteins up to two nodes to ascertain intimate protein interactions. Additional statistical analysis was performed to search for the top-ranked nodes which connected the largest number of other nodes. In this way, we were able to identify the intermediate hub proteins relevant to specific disorders. In our case, we reconstructed the PPI network by combining all six sub-networks and mapping to the reference network to identify candidates for common hubs (Figure 2B). After applying statistical degree analysis, we were able to identify several candidate hubs including EED, UBC, and KDM5A, which showed the highest degree statistics in the combined network. To verify that these common hubs were indeed specific for their respective neuronal disorders, we randomly chose 80 neuronal protein IDs from the Uniprot protein database for *Mus musculus* and created a PPI network using the same parameters and reference network. It was confirmed that EED was not indicated as the top-ranking hub protein in this random network. Thus, we concluded that changes in the hub proteins we identified here were indeed highly correlated with the six neurodegenerative diseases, as well as with 3OHB administration.

Our collective observations highlighted EED, one of the components of the Polycomb repressive complex 2 (PRC2) responsible for recognizing repressive trimethyllysine marks. We next tested the trimethylated lysine 27 of histone H3 using western blot analysis. As expected, the level of H3K27 trimethylation decreased along with the 3OHB treatment time, especially after 12 and 24 h of incubation, where it showed a dramatic drop upon treatment with 0.2 mM 3OHB (Figure 2C, left). 

At the same time, recent studies have shown that H3K4me3 also responds to 3OHB fluctuations and can rescue hippocampal memory defects in a mouse model of Kabuki syndrome [18]. In order to verify whether H3K4me3 could be altered in normal neurons, we next applied western blot analysis to detect the global alteration state of H3K4me3 in the cultured HT22 cells. Cell culture and treatment with 0.2 mM 3OHB was performed the same as described above. As shown in Figure 2C, H3K4me3 levels were clearly elevated in the HT22 cells upon 12 h of incubation with 0.2 mM 3OHB. In order to confirm this phenomenon in an animal model, we further analyzed both histone marks in fasted C57 mice. H3K27me3 was dramatically reduced in C57 mice after 24 h and H3K4me3 was elevated after 6 h of fasting (Figure 2D). This remarkable change in the entire brain tissue after fasting inspired us to further investigate the bivalent chromatin status of the central nervous system. Accordingly, the results demonstrated that both H3K27me3 and H3K4me3 are sensitive to 3OHB elevation both in cultured cells and in the brains of fasted mice.

### 2.4. The Correlation between Chromatin Bivalency and the Influence of 3OHB on Gene Expression Highlighted the 3OHB-Responsive Transcriptional Regulatory Network as an Intermediary between Disease-Related Genes and 3OHB

**Figure 3:**
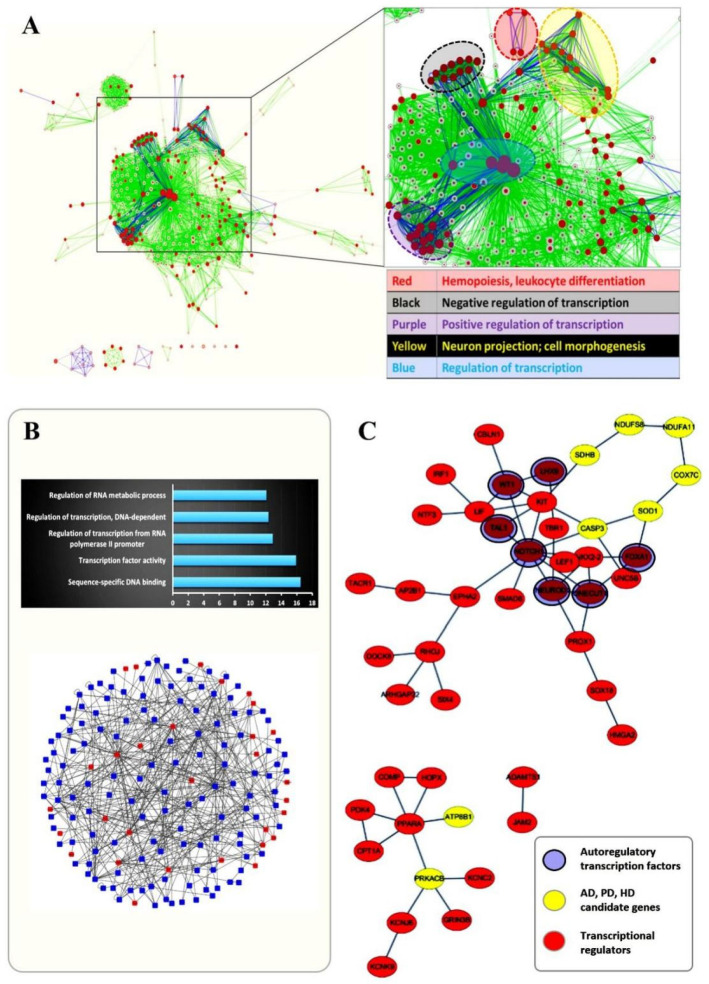
**The correlation between chromatin bivalency and 3OHB-perturbed gene expression patterns highlighted that the 3OHB-sensitive transcriptional regulatory network is a promoter of the interaction between disease-related genes and 3OHB.** (**A**) Biological processes associated with H3K27me3-H3K4me3 chromatin bivalency and processes perturbed by ketone bodies (1 mM 3OHB and 1 mM acetoacetate, 1 h) in neurons were extracted and overplayed to reveal possible correlations between chromatin bivalency and 3OHB. (**B**) Up, the overlapping genes were further subjected to gene ontology analysis; down, the protein-protein interaction network was seeded with the overlapping genes. Red nodes indicate seed genes and blue nodes represent intermediate protein-protein interactions between seed genes. (**C**) A network was generated using the STRING database by loading the overlapping genes as seeds which constitute nodes of a protein interaction network. Among them, yellow nodes represent genes related to neurodegenerative disease like AD, PD and HD. Autoregulatory transcription factors were highlighted with purple circles.

According to the western blot results, H3K4me3 and H3K27me3 were both sensitive to 3OHB, and may have altered the gene expression pattern in normal neurons upon 3OHB incubation. Thus, we used Chip-Seq and RNA-Seq data from previous research to decipher the correlation between chromatin bivalency and the 3OHB-perturbed gene expression pattern. The Chip-Seq (FPKM > 1.2) and RNA-Seq (*p* < 0.05) data were obtained from the study by [19] and GEO: GSE7323, respectively. They were first loaded into DAVID to collect annotation clustering information. Biological processes associated with H3K27me3-H3K4me3 bivalent chromatin and processes influenced by ketone bodies (1 mM 3OHB and 1 mM acetoacetate, 1 h) in neurons were overplayed to extract possible correlations in functional clustering. As shown in Figure 3A, the biological process groups shared between two different datasets were indicated with different colors after enrichment analysis. Obviously, gene transcription regulation processes were especially highlighted, and neuronal projection and differentiation processes were also relatively enriched. Additionally, according to the correlation analysis between our proteomics data and RNA-seq data from the references, metabolic genes related to neurodegenerative diseases were highlighted as exhibiting constant up- or downregulation both at the RNA and protein levels. Among them, the DNA methyltranferase DNMT3a and the H2B monoubiquitination regulator RNF20 were reported to directly participate in the maintenance of chromatin bivalency [20,21]. To further investigate the relevance of chromatin bivalency and the neuroprotective function of 3OHB, we constructed a network containing protein-protein and protein-DNA interactions using the Bisogenet plugin in Cytoscape. Seed proteins are indicated by red nodes and the interacting proteins or genes are labeled with blue nodes (Figure 3B). The truncated network is regulated by several key transcription factors (TFs), including Notch1, Gata4, Kit, Wt1, and Fox family proteins. We proposed that those transcription factors may be involved in the regulation mechanisms of genes related to neurodegenerative diseases that were altered by 3OHB. Thus, information on protein interactions between those two groups of genes was further extracted from the STRING database. As shown in Figure 3C, the disease-related genes formed a circular network that was connected with several key TFs. Particularly, seven TFs among them have been reported to form auto- or co-regulatory networks when the H3K27me3 state is changed [19]. This autoregulatory network co-occurred with networks of disease-related genes in a loop-like topology.

### 2.5. OHB Perturbed Chromatin Bivalency and Resulted in the Alteration of Neural Differentiation Processes

**Figure 4:**
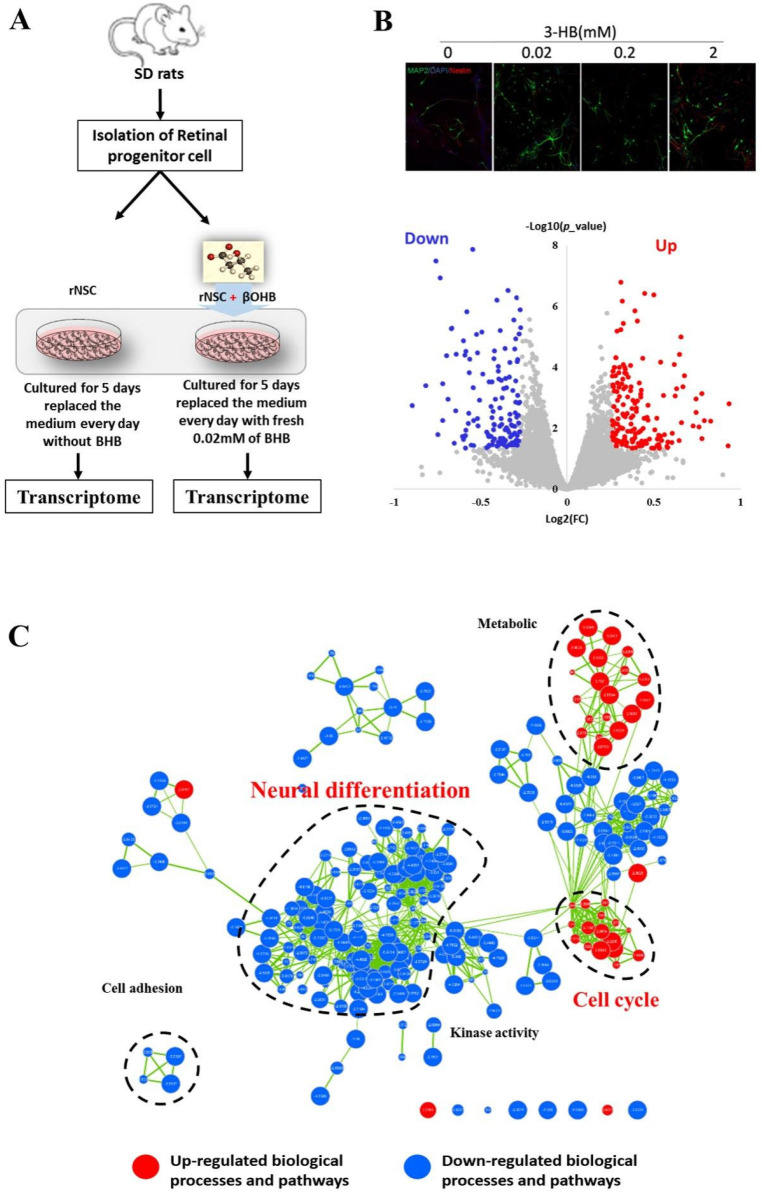
**BHB perturbed chromatin bivalency and resulted in the alteration of neural differentiation processes.** (**A**) Workflow of neural precursor cell isolation and 3OHB treatment for transcriptome analysis. (**B**) Up, immunocytochemical staining used to validate the alteration of the differentiation status of NSCs in the presence of 0.02, 0.2 and 2 mM 3OHB; down, volcano plot of the transcriptomic dataset showing changes of gene expression upon BHB administration ((5 days; *n* = 3 biological replicates) (*p* < 0.05; log2(fold change) > 0.25 or < −0.25. (**C**) GSEA extraction-enriched gene ontology map derived from the transcriptomic data. Red nodes indicate signaling pathways upregulated by 3OHB while blue nodes represent signaling pathways downregulated by it in NSCs. The cellular metabolic processes indicated by circles were increased by 3OHB treatment; biological processes related to neural differentiation were significantly inhibited (*p* < 0.001) while processes related to the cell cycle were significantly promoted by 3OHB (*p* < 0.001).

Another part of the genes exhibiting bivalent chromatin that were perturbed by 3OHB are involved in neuronal development. This phenomenon opens up the possibility of a novel and attractive application of 3OHB, in which it may be used to manipulate neural development processes. We consequently used transcriptomic profiling to evaluate whether the addition of low concentrations of 3OHB during NSC differentiation would be sufficient to affect neurodevelopmental processes. As described in the methods section, 0.02 mM 3OHB was added into the differentiation medium, and neuronal differentiation was induced for five days. Immunocytochemistry staining was used to detect and validate the alteration of the NSCs differentiation status. After five days of culture in differentiation medium, a part of the NSCs had differentiated into MAP2+ neurons. As expected, an alteration of the MAP2+ neuron population was observed upon 3OHB addition (Figure 4B up). The transcriptomes of differentiated cells from different groups were analyzed by RNA-seq (Figure 4A,B down). After global analysis of all differentially expressed genes and gene-set enrichment analysis, cellular metabolic processes were found to be increased by 3OHB, as expected. However, biological processes related to neural differentiation were significantly inhibited (*p* < 0.001), while cell-cycle-related processes were significantly promoted by 3OHB (*p*< 0.001) (Figure 4C). This result was in good agreement with the predictions from integrative analysis of the perturbation of chromatin bivalency. More importantly, 3OHB-mediated perturbation of NSC fate has to our best knowledge never been reported before, and this finding may be of great importance for investigations on how neuronal fate is manipulated by endogenous metabolites. This analysis thus demonstrates that the transcriptomic profile of differentiating neural stem cells was significantly altered by low concentrations of 3OHB.

### 2.6. Identification and Validation of Abundant Histone Lysine Hydroxybutyrylation (Kbhb) Sites

**Figure 5:**
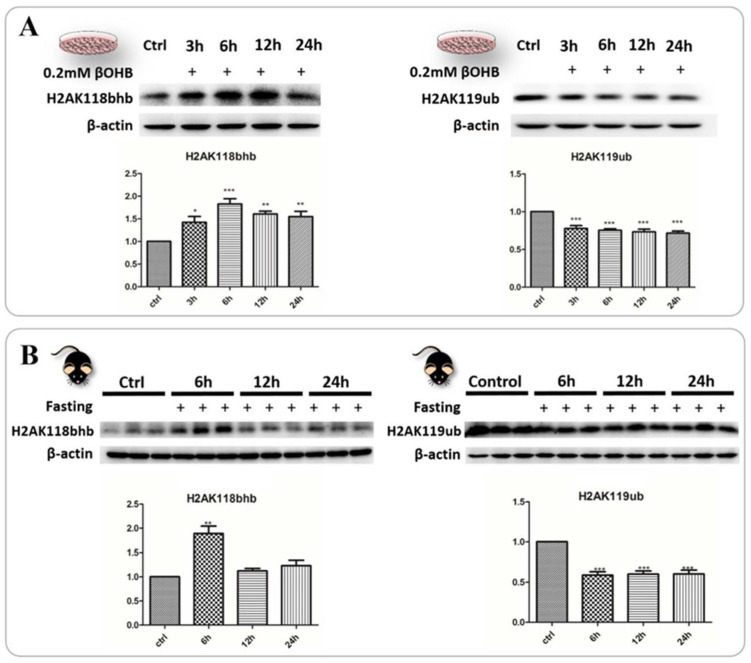
**Identification and validation of abundant histone lysine hydroxybutyrylation (Kbhb) sites.** (**A**) Western blot analysis (*n* = 3), left, revealed that 3OHB induced a significant increase of H2AK118bhb levels in the HT22 cells (at 6 h (*p* < 0.001), 12 h (*p* < 0.01) and 24 h (*p* < 0.01); right, H2AK119ub levels in the HT22 cells were significantly reduced after 3OHB treatment (*p* < 0.001). (**B**)Western blot analysis (*n* = 6), left, showing that fasting induced a significant increase of H2AK118bhb levels in C67 mice at 6 h of fasting (*p* < 0.01); right, H2AK119ub levels showed a similar pattern of significant reduction as observed in the HT22 cells (*p* < 0.001). Data are mean ± SEM *n* = 3 (**A**), Data are mean ± SEM *n* = 6 mice per group (**B**), one-way ANOVA, Tukey’s test, * means *p* < 0.05, ** means *p* < 0.01 while *** means *p* < 0.001 (**A**,**B**).

To extend this finding further and determine how 3OHB affects neuronal functions through epigenetic regulation, we re-evaluated the MS dataset using the Mascot search engine and identified several abundant epigenetic marks including H2AK118bhb and H3BK34bhb. Thus far, two of the most abundant histone lysine β-hydroxybutyrylation (Kbhb) sites have been confirmed to be tightly correlated with the administration of 3OHB and may be involved in the pathogenesis of neurodegenerative diseases. Interestingly, the two Kbhb sites H2AK118bhb and H3BK34bhb are also the most well-defined histone modifications and are required for other modifications including H3K4 and H3K79 methylation (requires H2BK34ub1) and H3K27 trimethylation (requires H2AK119ub1). Accordingly, we reasoned that these two monoubiquitination sites, which also may potentially be occupied by hydroxybutyrylation signals, may dramatically affect the H3K4, H3K27 and H3K79 methylation levels. This theory perfectly matched our previous proteomic assessment of the critical role of H3K27me3 and H3K4me3 in the cellular response to 3OHB. More importantly, the fluctuations of the H3K4me3 level due to 3OHB administration were also indicated in a mouse model of Kabuki syndrome [18]. To gain insights into whether histone lysine hydroxybutyrylation responds to the addition of 3OHB or prolonged fasting, and how it can further cause alterations in the levels of histone lysine monoubiquitination, we performed immunoblot analysis to determine the global levels of H2AK118bhb and H2AK119ub1 both in HT22 cells and in brain samples. As shown in Figure 5, H2AK118bhb was dramatically elevated upon 3OHB treatment in normal neurons, while H2AK119ub1 was dramatically decreased under the same conditions, and a similar pattern was observed in brain samples from fasted C57 mice. H2AK118 and H2AK119 are vicinal lysine residues and have been considered as one of the most important monoubiquitination sites associated with transcriptionally silent facultative heterochromatin [22,23]. We reasoned that H2AK118bhb was able to directly occupy the monoubiquitination sites or sterically hinder the monoubiquitination reaction on H2AK119. Although histone lysine hydroxybutyrylation has been identified on multiple sites, and our proteomics data were not able to cover all of those sites, H2AK118bhb1 and H2BK34bhb1 can nevertheless be considered as highly abundant lysine hydroxybutyrylation sites on histones, and consequently may be involved in critical biological functions related to the histone methylation profile.

## 3. Discussion

Ketogenic diets have been applied control the progression of several neuronal disease conditions, including epilepsy, autism, and neurodegenerative diseases [24,25]. Along with successful clinical trials, ketogenic diets have been demonstrated to have neuroprotective effects and possible applications in other neuronal diseases including AD, PD, ALS, and their detailed mechanism of action has also been investigated for decades [15].

As mentioned above, biological processes related to neurodegenerative diseases were enriched and highlighted in our proteomics study, which was also in agreement with previous research performed on neuronal cells and experimental animals [2,5]. Reactive oxygen species and oxidative damage have been considered to be major contributors to the pathogenesis of neurodegenerative diseases [26]. However, the specific relationships between 3OHB, HDACi and neuroprotection have not been fully elucidated or understood at the systems level. Therefore, we used known protein networks related to neurodegenerative diseases as a matrix integrated with our proteomics dataset to further investigate the protein-protein interaction networks perturbed by 3OHB in normal neurons. PPIs perturbed by 3OHB were generated to search for the perturbed hub proteins that were shared by all six neurodegenerative diseases. H3K27me3, which is a substrate of the EED protein, was uncovered as a putative perturbed translational hub in 3OHB-related neuroprotective functions.

H3K27me3 has been extensively studied in neurons, and those studies indicated that its levels dramatically correlated with neurodegenerative diseases [19,27,28]. Based on our biochemical analysis, the global level of H3K27me3 in HT22 cells was dramatically altered by 0.2 mM 3OHB treatment. Considering the importance of H3K27me3 for critical functions in neural stem cell differentiation and adult neuron specification maintenance, this phenomenon strongly suggests that 3OHB may interfere with coordinated transcriptional processes that trigger regulatory pathways involved in neurodegeneration, and further involve the functional dysregulation of vulnerable or unhealthy neuronal networks.

Polycomb group (PcG) proteins, which include the PRC1 and PRC2 complexes, are critical epigenetic repressors responsible for transcriptional regulation of cell differentiation and development [29]. PRC1 and PRC2 catalyze specific histone modifications including H2A lysine 119 ubiquitylation (H2AK119u1) and H3 lysine 27 methylation (H3K27me3), respectively [30]. PRC1-dependent H2AK119ub1 can lead to the recruitment of PRC2 complexes to H3K27me3, which initiates polycomb domain formation [22,31,32,33]. In this scenario, H2AK118/119ub formation is critical for H3K27me3 accumulation. Accordingly, we reasoned that 3OHB-induced H3K27me3 fluctuations at the global level may be caused by H2AK118/119bhb alteration, since we found that H2AK118/119 and H2BK34 are the most abundant hydroxybutyrylation sites in the histones of normal neurons. It is possible that endogenous H2AK118/119bhb may be involved in H2AK118/119ub-dependent H3K27me3 formation or even the catalysis of H2AK118/119ub itself. Thus, our findings indicate the possibility of an additional role of H2AK118/119bhb in polycomb domain formation and transcriptional regulatory circuitry.

At the same time, H2BK34 monoubiquitination has also been proved to be the major regulator of H3K4 and H3K79 methylation [34,35,36]. After measuring the global H3K4me3 level by western blot analysis, a time-dependent fluctuation pattern appeared that was comparable with that of H3K27me3. H3K4me3 is another critical histone marker for neuronal health and function [37,38,39], and it is sharply enriched at gene-proximal promoters and transcription start sites (TSS) [40,41]. Recent findings demonstrated that H3K4me3 in the human prefrontal cortex is highly regulated in a cell type and subject specific manner, which highlights age-correlated neuronal chromatin remodeling, with important implications for neurodevelopmental disorders [41], or neurodegenerative diseases [42,43]. The simultaneous presence of H3K4me3 and H3K27me3 at a single locus was defined as a bivalent chromatin state, i.e., the occurrence of histone modifications corresponding to gene activation (trimethylated H3K4) and PRC2-mediated gene repression (trimethylated H3K27). Chromatin bivalency has been recognized in ESCs during epigenetic regulation of development, and this epigenetic feature has been interpreted as conferring pluripotency on ESCs [44,45]. Our results not only confirm the sensitivity of H3K4me3 and H3K27me3 in normal neurons to 3OHB fluctuations, but also support a new theory in which chromatin bivalency can be perturbed by 3OHB in the normal nervous system. In particular, the selective changes of gene expression reflect a particular group of 3OHB-perturbed bivalent genes enriched for transcriptional regulation that are related to neuronal functions and especially neurodegenerative diseases, or critical signaling components involved in those conditions (Figure 3C). However, when comparing three datasets, only three bivalent genes were found to overlap, and their mRNA and protein alterations were inconsistent. It is possible that the neuronal responses to 3OHB and other ketone bodies are mediated by the temporary perturbation of chromatin bivalency rather than by a permanent promotion of bivalent gene fluctuation. In support of this idea, several transcription factors sensitive to ketone bodies have been shown to have potential auto-regulatory functions [19]. We therefore propose that those transcription factors form auto-regulatory feedback loops that stabilize fundamental processes and mediate the sensitivity to various environmental factors including metabolic fluctuations.

Based on this hypothesis, we performed NSC differentiation experiments to confirm that 3OHB is actually able to perturb the NSCs differentiation process. As we expected, both immunocytochemical staining and transcriptomic analysis have clearly indicated that 3OHB was able to inhibit neural differentiation and promote the cell cycle. In addition to the data from “omics” analysis, both H3K27me3 and H3K4me3 were sensitive to low concentrations of 3OHB, which makes them possible mediators of neuroprotection, and likely promoters of neural differentiation. Thus, one can envision a scenario in which the presence of 3OHB may trigger an enzymatic reaction and further occupy histone tail monoubiquitination sites such as H2AK118/119 and H2BK34. Such a scenario may also entail the perturbation of the monoubiquitination pattern, which would further lead to alterations of monoubiquitination-dependent histone methyltransferase recruitment efficiency [22,23,31,33,34,46,47]. Although the alteration of hydroxybutyrylation and monoubiquitination of H2AK119 has also been observed upon 3OHB administration, it is not sufficient to confirm that the response of H3K27me3 and H3K4me3 to 3OHB administration is dependent on H2AK118/119 and H2BK34 hydroxybutyrylation. Nevertheless, the induction of chromatin bivalency by 3OHB and other ketone bodies, and the fluctuation of the related transcription factors implies a scenario in which ketone-body-dependent neuroprotection may be mediated by histone modifications and alterations of chromatin structure. To this end, our results build a connection between ketone bodies, a bivalent chromatin state, DNA methylation, neurodegenerative diseases, and neural differentiation.

## 4. Materials and Methods

### 4.1. HT22 Cell Culture and 3OHB Treatment

Murine hippocampal HT22 cells were purchased from Hongshun Biologicals (Shanghai, China). The details of HT22 cell culture and 3OHB treatment were included in Supplemental Experimental Procedures.

### 4.2. Culture of NSCs and Transcriptome Sequencing

NSCs were isolated from the cerebral cortex of rat embryos on embryonic day 14.5 (E14.5) and cultured in serum-free growth medium as described previously [48]. The primary isolated cells were subcultured every five days and P3 cells were used for transcriptome sequencing. See Supplemental Experimental Procedures for details.

### 4.3. Animal Maintenance and Treatment

All procedures followed the Guide for the Care and Use of Laboratory Animals: Eighth Edition (ISBN-10: 0-309-15396-4), and the animal experiment protocol was approved by the animal ethics committee of Xi’an Jiaotong University’s School of Life Science and Technology (approval No. SCXK (陕) 2017-003). The details of animal maintenance and treatment were included in Supplemental Experimental Procedures.

### 4.4. Mass Spectrometry of Proteins

Protein extraction was performed using approximately 1 × 10^7^ cells per group. Proteins were digested into peptides and labeled via Isobaric Dimethylation as before [48]. The peptides were then subjected to LC-MS/MS. See also Supplemental Experimental Procedures for details.

### 4.5. Statistical Analysis

Statistical significance was determined using single-factor analysis of variance (one-way ANOVA) and Tukey’s range test via GraphPad Prism V7.0 (GraphPad Software, USA). The results were considered significant when the *p*-value was less than 0.05.

## Supplementary Materials

The following supporting information can be downloaded at: https://www.mdpi.com/article/10.3390/ijms24010868/s1, Supplemental Information includes supplemental experimental procedures, Supplemental Table S1 and Supplemental Table S2. Refs. [14,48,49,50,51,52,53,54,55,56,57,58] are cited in Supplementary Materials.
