# Supplementary material for: Integrative Proteome Analysis Revels 3-Hydroxybutyrate Exerts Neuroprotective Effect by Influencing Chromatin Bivalency
Source: Int J Mol Sci. 2023 Jan 3;24(1):868. doi: 10.3390/ijms24010868 (PMC9821512; doi:10.3390/ijms24010868)
Supplement: Supplementary file 1 [file ijms-24-00868-s001.zip › Supplemental Experimental Procedures.pdf]

SUPPLEMENTAL EXPERIMENTAL PROCEDURES

HT22 Cell Culture and 3OHB Treatment

Murine hippocampal HT22 cells were purchased from Hongshun Biologicals (Shanghai, China) and cultured in high-glucose Dulbecco's modified Eagle's medium (DMEM) supplemented with 10% fetal bovine serum (FBS, HyClone, GE Healthcare, USA), 100 U/ml penicillin (Beyotime, China) and 100 μg/ml streptomycin (Beyotime, China) at 37 ºC, in a humidified incubator with 5% CO_2. 0.2mM 3OHB (98%, SIGMA, USA) was freshly dissolved in the cell culture medium. Either 3OHB-containing medium or regular medium was applied for cell culture, which was continued for 3, 6 or 12 hours before protein extraction, as indicated.

Culture of NSCs and Transcriptome Sequencing

NSCs were isolated from the cerebral cortex of rat embryos on embryonic day 14.5 (E14.5) and cultured in serum-free growth medium as described previously (Lu et al., 2011, 2013). The primary isolated cells were subcultured every 5 days and P3 cells were used for transcriptome sequencing. Briefly, 0.02 mM 3OHB was included in the PBS, the half amount of 3OHB was added into the medium after 24 hours of culture for the 3OHB-treated group, and the medium was replaced after 48 hours of culturing for both the control and the 3OHB-treated groups. NSCs were cultured in differentiation medium [DME/F-12(GE Healthcare Life Science, USA)+1*B27(GE Healthcare Life Science, USA)+1*N2(GE Healthcare Life Science, USA)+1%FBS（Gibco, USA）for five days for both the control and the 3OHB treatment groups. Total RNA was extracted from each sample using TRIzol Reagent (Life technologies, USA) and the preparation of whole-transcriptome libraries and deep sequencing were conducted as described before (1).

Immunocytochemical Staining

Immunocytochemical staining was performed as described previously (2). For the identification of NSCs, cell aggregates were seeded onto poly-L-lysine (PLL) -coated coverslips (SIGMA, USA). Before staining, all cells were fixed with 4% paraformaldehyde (PFA; Biosharp, China) for 12 hours at 4 ºC. Primary monoclonal mouse anti-nestin (1:500, Millipore, Temecula, CA, USA) and monoclonal mouse anti-MAP2 (1:500, Millipore, Temecula, CA, USA) antibodies were used to identify NSCs and neurons, respectively. All primary antibodies used in this study were diluted in PBS with 2% normal goat serum (NGS; Biosharp, China). The blocking solution contained 5% NGS and 0.25% Triton X-100 in PBS. DyLight 488 and DyLight 594-conjugated goat anti-rabbit IgG or anti-mouse IgG (1:1000; Abbkine, Wuhan, China) were used as secondary antibodies. Cell nuclei were counterstained with DAPI-containing mounting media (Vector, Burlingame, CA, USA) and

visualized under a BX57 fluorescence microscope (Olympus, Tokyo, Japan) equipped with a DP70 digital camera and the DPManager software. For the negative control, the primary antibody solution was replaced with blocking buffer.

Animal Maintenance and Treatment

All procedures followed the Guide for the Care and Use of Laboratory Animals: Eighth Edition (ISBN-10: 0-309-15396-4), and the animal experiment protocol was approved by the animal ethics committee of Xi'an Jiaotong University's School of Life Science and Technology (approval Nr. SCXK(陕）2017-003). Five-week-old male and female C57BL/6 (c57) mice (n=12 for each sex) were purchased from SLAC Laboratory Animal Co. Ltd. (Shanghai, China). The mice were housed at a temperature between 22–25 ºC at 60% humidity on a 12-h light/dark cycle with free access to food and water before the experiment. The mice were divided into four groups of six animals each, which consisted of three females and three males in different cages. The mice were acclimatized for three days before entering the fasting protocol. The control mice were immediately sacrificed by rapid decapitation performed by an adequately trained person. Other mice were fasted for 6, 12 and 24 hours before sacrificing. Water was supplied ad libitum throughout the experiment for all animals. The brains were dissected immediately, frozen in liquid nitrogen, and stored at −80 ºC until further use.

Sample Preparation for LC-MS/MS

Protein extraction was performed using approximately 1×10^7 cells per group. The HT22 cells were washed twice with ice-cold PBS, and then lysed in 1.0 ml of urea lysis buffer (8 M urea, 20 mM Tris-HCl (pH 7.4), 10 mM EDTA, 0.15M NaCl, 1% Octyl β-D-glucopyranoside (SIGMA, USA), 1 × EDTA free cocktail (Roche, UK)). Cell lysates were vigorously mixed for 10 min at room temperature and further sonicated for 20s (20 kHz, 30% amplitude). Proteins were reduced by freshly adding 10 mM DTT (Aladdin, China) to break disulfide bonds, and incubated in the dark for 1 hour at 37 ºC, followed by alkylation with 40 mM iodoacetamide (SIGMA, USA) for 30 min at room temperature. The lysates were diluted using 50 mM NH_4HCO_3 to yield a urea concentration of 1M. Proteins were digested at 37 ºC overnight after adding sequencing-grade trypsin (V5111, Promega, USA) into the diluted protein solutions at a ratio of 1:50 (trypsin:protein - w:w). The digested solutions were acidified by adding 5% formic acid and further desalted using an HLB column (Waters Corporation, United Kingdom). The eluate (elution buffer: water/acetonitrile/formic acid = 20:80:0.1) was evaporated to dryness in a vacuum centrifuge.

Labeling of Peptides via Isobaric Dimethylation

Isobaric dimethylation of N–termini and lysine amino groups with HCHO ($D^{13}CDO$; SIGMA, USA) and $NaBH_3CN$ (SIGMA, CHINA) was carried out using the same protocol as reported before (3). After labeling, the isobaric peptide solution with light (+28) or heavy (+34) labels for both the control and the 3OHB treatment, respectively were mixed at a ratio of light/heavy=1:1 (control (light): treatment (heavy)) as forward labeling and 1:1 (treatment (light): control (heavy)) as reciprocal labeling, and freeze-dried for subsequent HPLC-SCX fractionation.

HPLC-SCX Fractionation of Dimethyl Labeled Mixed Peptides.

SCX chromatography was performed on an Åkta Purifier modular liquid chromatography system (GE Healthcare, USA) at a flow rate of 1 ml/min using the SCX mobile phase. Both 254 and 280 nm UV spectra were used to monitor the effluent. A polysulfoethyl A column (4.6 × 200 mm, 5 μm, 200 Å, PolyLC, USA) was equilibrated with 0.01% H_3PO_4, pH 3.0 (w/v) in 25% acetonitrile. The dimethyl-labelled peptide mixtures were dissolved in 500 μL of buffer A, and loaded onto the column. After 5 min, 5 mL of 10 mM KH_2PO_4 pH 3.0 in 25% acetonitrile (buffer solution A) was injected. A 25-min gradient was run from 0% to 25% of buffer solution B (10 mM KH_2PO_4, 300 mM KCl, pH 3.0 in 25% acetonitrile), followed by an increase to 100% of buffer B in 25 min, and finally 5 min of isocratic elution with 100% buffer B. Fractions were collected every minute and every 6 adjacent fractions from No. 1 to No. 60 were combined, desalted and freeze-dried. The amount of peptide in each fraction was measured using the protein Micro BCA Protein Assay Kit (Thermo Fisher Scientific, China) with BSA as the protein standard.

LC-MS/MS Analysis

For liquid chromatography–tandem mass spectrometry (LC–MS/MS) analysis, the peptides were separated using a 120-min gradient on a fused silica capillary analytical column (75 μm ID, 150 mm length; Upchurch, Oak Harbor, WA) packed with C18 resin (300 Å, 5 μm, Varian, Lexington, MA) on a NanoLC system (Proxeon, Denmark). The elution was achieved at a flow rate of 0.25 μL/min using the following gradient: 0–8 min 2% B, 8–12 min 2–10% B, 12–90 min 10–50% B, 90–100 min 50-98% B and 100-120 min 98-2% B (acetonitrile/formic acid, 100:0.1). The analytical column was directly interfaced with a Thermo Scientific Q Exactive mass spectrometer which was operated in the data-dependent acquisition mode with a full-scan mass spectrum in the Orbitrap (300–1800 *m/z*, 75 000 resolution). 10 of the most abundant m/z signals were chosen from the primary scan for collision-induced dissociation at 32% normalized collision energy (HCD). The mass window for precursor ion selection was 2.0 *m/z*, the threshold for triggering MS/MS experiments was 8.0×10^3, and the dynamic exclusion time was 20 s.

Proteomics Data Processing

The collected RAW data files were analyzed using the MaxQuant software, version 1.5.3.23 (Cox and Mann, 2008). Protein identification was achieved using the Andromeda search engine (Cox et al., 2011) which was included in the MaxQuant software. The parameters from MaxQuant were set to search against the UniProt *Rattus* database (UniProt release 2015_10). Isobaric labelling with light (+28 Da) and heavy (+34 Da) dimethyl labels on N-termini and lysine residues was chosen as the isobaric labeling method. The oxidation of methionine and carbamidomethylation of cysteine were set as the variable modification and fixed modification, respectively. Up to two missed trypsin cleavages were allowed. Peptides were searched using an MS1 tolerance of 6 ppm and MS2 tolerance of 1.5 Da. The false discovery rate (FDR) was 0.1% for the identification of peptides and proteins. Protein quantitation was performed using unmodified peptides. The quantitative protein and peptide output from MaxQuant was analyzed using Perseus (version 1.5.3.2). In addition to the default FDR thresholds in MaxQuant, additional cleaning procedures were used to eliminate the proteins with less than 2 identified peptides, contaminants and reverse hits.

Transcriptomics Data Processing

Filtering raw reads as well as mapping and identification of differentially expressed genes was the same as described previously (4). Differentially expressed genes were selected according to the FDR threshold (FDR < 0.05) using the DESeq2 package from Bioconductor (Cambridge, MA, USA) from control and 3OHB-treated NSCs (4).

Bioinformatics

From the four biological replicates that were generated for each condition and treatment mixture (Table S1), we filtered the protein list by keeping proteins that were successfully identified and quantified in three out of four replicates. We then applied a log2 transformation to the fold-change data to obtain the protein abundance difference for each protein.

The Gene Set Enrichment Analysis (GSEA) tool (5) was used to find enriched biological processes and pathways according to the product manual, using default parameters. These pathways were then visualized using the Cytoscape (3.4.0) plug-in EnrichmentMap.

BisoGenet plugin (6) was used to construct the protein–protein interaction networks. All protein IDs generated from our proteomics study were matched against the gene lists of the six types of disease: the ASD, epilepsy and Alzheimer’s disease gene lists from (7), ALS by combining references (8, 9), Parkinson’s disease from PDmap and UK PD project (10, 11) and Huntington's disease from (12). Data processing and

preparation were performed using Microsoft Excel 2010. The resulting disease-related protein IDs were used as seeds for mapping the protein-protein interaction database from the BioGRID and MINT databases to generate a 3OHB-perturbed disease network using the BisoGenet plugin. The BisoGenet parameters were as follows: neighbors to seed proteins was set up to a distance of 2, the output network was represented as proteins; degree, betweenness and closeness were used to perform network statistical analysis. The degree statistic was used to further identify the hub proteins.

Western Blot Analysis

HT22 cells were cultured in 6-well plates (Eppendorf, CHINA) in DMEM medium with 10% FBS to a density of 2×10^5 cells/well. 3OHB was added to the medium at a final concentration of 0.2 mM for the indicated time. Whole-cell lysates were extracted the same as described for the proteomics sample preparation. Mice brain tissue was homogenized in urea lysis buffer and homogenates were centrifuged at 13 000 × *g* at 4ºC for 20 min. Proteins were separated via SDS/PAGE on a 12% acrylamide gel, and electro-blotted onto Hybond-P PVDF membranes (GE Healthcare, USA). The membranes were subsequently blocked using TBST with 5% (w/v) BSA (bovine serum albumin; Sigma-Aldrich, UK) for 2 h at room temperature, and then incubated at 4°C overnight with the primary antibodies in TBST with 5% (w/v) BSA. The primary antibodies were used as follows: rabbit anti-H3K27me3 (1:1000; Millipore, Cat.#07-449), anti-H3K4me3 (1:1000; Cell Signaling technology, #9751) and anti-H2AK119ub (1:1000; Cell signaling technology, #8240, USA), anti-H2AK118bhb (1:1000; PTMlab, China) or anti-β-actin (1:1000; Bioss, China). After washing with TBST (3 times, 10 min each), the membranes were incubated with the horseradish peroxidase (HRP)-conjugated secondary anti-rabbit antibody (Beyotime, China), which was diluted 1:5000. After washing with TBST (3 times, 10 min each), the target protein bands were detected using the Immobilon™ Western Chemiluminescent HRP Substrate (Millipore, USA) and visualized using a Chei Scope 3300 Mini (CLINX, China).

Statistical Analysis

Statistical significance was determined using single-factor analysis of variance (one-way ANOVA) and Tukey's range test via the PrismDemo software (GraphPad Softwar, USA). The results were considered significant when the *p*-value was less than 0.05.

References

1. Wang, W., Meng, M., Zhang, Y., Wei, C., Xie, Y., Jiang, L., Wang, C., Yang, F., Tang, W., and Jin, X. (2014) Global transcriptome-wide analysis of CIK cells identify distinct roles of IL-2 and IL-15 in acquisition of cytotoxic capacity against tumor. *BMC medical genomics* 7, 49

2. Jiao, Q., Li, X., An, J., Zhang, Z., Chen, X., Tan, J., Zhang, P., Lu, H., and Liu, Y. (2017) Cell-Cell Connection Enhances Proliferation and Neuronal Differentiation of Rat Embryonic Neural Stem/Progenitor Cells. *Frontiers in cellular neuroscience* 11, 200

3. Boersema, P. J., Raijmakers, R., Lemeer, S., Mohammed, S., and Heck, A. J. (2009) Multiplex peptide stable isotope dimethyl labeling for quantitative proteomics. *Nature protocols* 4, 484-494

4. Love, M. I., Huber, W., and Anders, S. (2014) Moderated estimation of fold change and dispersion for RNA-seq data with DESeq2. *Genome biology* 15, 550

5. Subramanian, A., Tamayo, P., Mootha, V. K., Mukherjee, S., Ebert, B. L., Gillette, M. A., Paulovich, A., Pomeroy, S. L., Golub, T. R., and Lander, E. S. (2005) Gene set enrichment analysis: a knowledge-based approach for interpreting genome-wide expression profiles. *Proceedings of the National Academy of Sciences* 102, 15545-15550

6. Martin, A., Ochagavia, M. E., Rabasa, L. C., Miranda, J., Fernandez-de-Cossio, J., and Bringas, R. (2010) BisoGenet: a new tool for gene network building, visualization and analysis. *BMC bioinformatics* 11, 91

7. Niere, F., Namjoshi, S., Song, E., Dilly, G. A., Schoenhard, G., Zemelman, B. V., Mechref, Y., and Raab-Graham, K. F. (2016) Analysis of proteins that rapidly change upon mechanistic/mammalian target of rapamycin complex 1 (mTORC1) repression identifies parkinson protein 7 (PARK7) as a novel protein aberrantly expressed in tuberous sclerosis complex (TSC). *Molecular & Cellular Proteomics* 15, 426-444

8. Abel, O., Powell, J. F., Andersen, P. M., and Al‐Chalabi, A. (2012) ALSoD: A user‐friendly online bioinformatics tool for amyotrophic lateral sclerosis genetics. *Human mutation* 33, 1345-1351

9. Mao, Y., Kuo, S.-W., Chen, L., Heckman, C., and Jiang, M. (2017) The essential and downstream common proteins of amyotrophic lateral sclerosis: A protein-protein interaction network analysis. *PloS one* 12, e0172246

10. Fujita, K. A., Ostaszewski, M., Matsuoka, Y., Ghosh, S., Glaab, E., Trefois, C., Crespo, I., Perumal, T. M., Jurkowski, W., and Antony, P. M. (2014) Integrating pathways of Parkinson's disease in a molecular interaction map. *Molecular neurobiology* 49, 88-102

11. Foulger, R. E., Denny, P., Hardy, J., Martin, M. J., Sawford, T., and Lovering, R. C. (2016) Using the gene ontology to annotate key players in Parkinson’s disease. *Neuroinformatics* 14, 297-304

12. Stroedicke, M., Bounab, Y., Strempel, N., Klockmeier, K., Yigit, S., Friedrich, R. P., Chaurasia, G., Li, S., Hesse, F., and Riechers, S.-P. (2015) Systematic interaction network filtering identifies CRMP1 as a novel suppressor of huntingtin misfolding and neurotoxicity. *Genome research* 25, 701-713
